# Peritumoural Strain Elastography of Newly Diagnosed Breast Tumours: Does Maximum Peritumoural Halo Depth Correlate with Tumour Differentiation and Grade?

**DOI:** 10.3390/diagnostics13122064

**Published:** 2023-06-14

**Authors:** Leonhard Gruber, Johannes Deeg, Daniel Egle, Afschin Soleiman, Valentin Ladenhauf, Anna Luger, Birgit Amort, Martin Daniaux

**Affiliations:** 1 Department of Radiology, Medical University Innsbruck, Anichstraße 35, 6020 Innsbruck, Austria; leonhard.gruber@i-med.ac.at (L.G.);; 2 Department of Obstetrics and Gynaecology, Medical University Innsbruck, Anichstraße 35, 6020 Innsbruck, Austria; 3 Institute for Pathology, INNPath, University Hospital Tirol Kliniken, Anichstraße 35, 6020 Innsbruck, Austria

**Keywords:** strain elastography (SE), peritumoural halo depth, breast tumour, St. Gallen Ki67

## Abstract

To evaluate the diagnostic utility of the maximum ultrasound strain elastography (SE) halo depth in newly diagnosed and histologically confirmed breast lesions, a retrospective study approval was granted by the local Ethical Review Board. Overall, the maximum strain elastography peritumoural halos (SEPHmax)—the maximum distance between the SE stiffening area and the B-mode lesion size—in 428 cases with newly diagnosed breast lesions were retrospectively analysed alongside patient age, affected quadrant, tumour echogenicity, size, acoustic shadowing, and vascularity. Statistical analysis included an ordinary one-way ANOVA to compare the SEPHmax between BI-RADS 2, 3, and 5 groups and between tumour grades 1, 2, and 3. A binary regression analysis was used to determine the correlation between tumour malignancy and the above-mentioned demographic and imaging factors. SEPHmax was significantly higher in BI-RADS 5 tumours (5.5 ± 3.9 mm) compared to BI-RADS 3 (0.9 ± 1.7 mm, *p* < 0.0001) and 2 (0.6 ± 1.4 mm, *p* < 0.0001). The receiver operating characteristic area under the curve was 0.933 for the detection of BI-RADS 5 lesions. Furthermore, tumour grades 2 (5.6 ± 3.6 mm, *p* = 0.001) and 3 (6.8 ± 4.2 mm, *p* < 0.0001) exhibited significantly higher SEPHmax than grade 1 tumours (4.0 ± 3.9 mm). Similarly, St. Gallen Ki67-stratified low-risk (*p* = 0.005) and intermediate-risk (*p* = 0.013) tumours showed smaller SEPHmax than high-risk tumours. Multivariate analysis revealed a significant correlation between malignant differentiation and SEPHmax (standardized regression coefficient 3.17 [95% confidence interval (CI) 2.42–3.92], *p* < 0.0001), low tumour echogenicity (1.68 [95% CI 0.41–3.00], *p* = 0.03), and higher patient age (0.89 [95% CI 0.52–1.26], *p* < 0.0001). High SEPHmax is a strong predictor for tumour malignancy and a higher tumour grade and can be used to improve tumour characterisation before histopathological evaluation. It may also enable radiologists to identify lesions warranting observation rather than immediate biopsy.

## 1. Introduction

Breast cancer is the most common malignancy in women at an incidence of up to 92 per 100,000 in highly developed countries, which keeps rising due to an increase in life expectancy [1]. Fortunately, up to 80% of early stage, non-metastatic cases can be cured [2]. Advanced breast cancer, defined by distant metastasis, is still considered incurable [2], underlining the importance of early detection and treatment.

Women are encouraged to take part in regular mammography programmes to detect carcinomas at an early stage and to enable early and curative treatment. Such programmes have led to a relative reduction in mortality of up to 20% [3]. Necessary treatment extent may not be impacted to such a degree, however [2,4].

Even though ultrasound detection rates in combination with conventional mammograms can reach substantial values of up to 92.6 to 94.4% [5], differentiation between benign and malignant tumours can be difficult at a reported correct classification rate of approximately two-thirds of tumours [5,6,7].

Tissue elasticity assessment—elastography—has seen an increase in clinical interest over the last decade, as increased tissue stiffness has been linked to high cellularity and malignancy in some entities [8]. While shear wave elastography is considered a quantitative method, as a shear wave pulse is generated, and the consecutive tissue deformation along the vertical wave propagation is measured and expressed as a pulse propagation speed or tissue elasticity [9,10], it is fraught with differing vendor-specific quantification algorithms and high susceptibility to applied pressure during measurement [11]. In strain elastography (SE), on the other hand, the examiner repeatedly exerts controlled pressure on the lesion via the ultrasound transducer [12]. Relative tissue displacement is then mapped based on an in-machine pixel-shift evaluation [13], allowing for visual assessment of lesion stiffness, such as the Tsukuba score or a semiquantitative ratio of lesion and fat tissue [14]. It is known that malignancies tend to present with a size discrepancy between B-mode and elastogram presentation, potentially appearing larger on the elastogram image. This appearance was termed a “halo” [10]. This apparent hardening of the peritumoural space is likely based on desmoplastic and fibrotic changes representing a reaction to tumour infiltration [15,16,17,18].

The aims of this retrospective study were to assess the diagnostic utility of the maximum ultrasound strain elastography (SE) halo depth in newly diagnosed breast lesions and to assess whether the maximum strain elastography peritumoural halo (SEPHmax) correlates with tumour differentiation, tumour grades, and (immuno)histology findings.

## 2. Materials and Methods

### 2.1. Approval by the Ethical Review Board (ERB)

Study approval for this retrospective study was granted by the Ethical Review Board on 21 July 2021 (ERB proposal 1224/2021).

### 2.2. Inclusion Criteria and Screening Procedure

**Figure 1:**
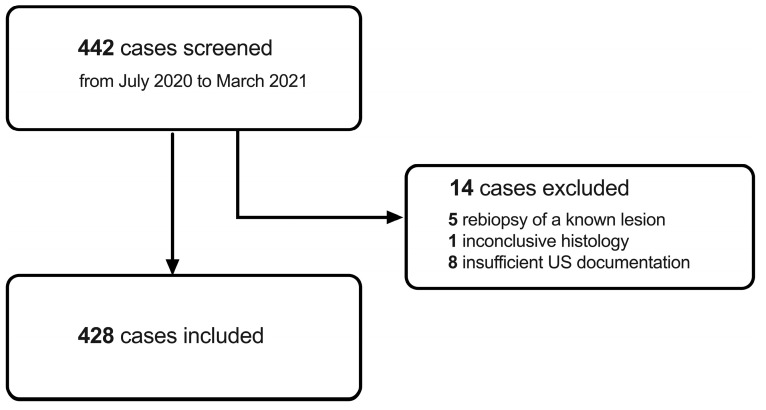
Overview of participant screening, exclusion, and inclusion.

All women having undergone an ultrasound-guided biopsy of an unknown breast lesion from 1 September 2020 to 31 March 2021 were retrospectively screened. For an overview of the inclusion criteria, please refer to Table 1. Among 442 women screened, 14 were excluded due to re-biopsy of a known lesion (*n* = 5), inconclusive histology (*n* = 1), or insufficient ultrasound documentation (*n* = 8) (Figure 1). Accordingly, 428 women could be included for further analysis.

### 2.3. Ultrasound Examination Procedure

All women in our second-level centre undergo a standardized ultrasound examination if referred for (1) routine or screening mammography in cases of breast density ACR C or D, or in case of (2) a suspicious finding upon mammography, (3) referral due to a suspicious finding upon clinical examination, or (4) referral from an extramural radiology practice due to suspicious imaging findings. Examinations were performed on an Acuson Sequoia, Acuson S2000 or S3000 Evolution US scanner with an 18–6 or 13.5 MHz high-resolution probe (Siemens, Erlangen, Germany) encompassing a systematic scanning of the axillary and breast regions. Focal lesions were documented in two perpendicular planes to determine the relation to surrounding tissue, size, B-mode, and Doppler properties. SE was performed by repeated controlled compression of the tumour and the surrounding tissue. Compression quality was assessed by visual inspection and by the vendor-specific quality index. Only SE images with a high quality index (>90 on Acuson S2000 or S3000, >65 on Acuson Sequoia) were used for further analysis. In addition, special attention was paid to ensure that the elastographic ROI was no more than a quarter in the chest wall in order to avoid any artefacts. Based on previously published criteria, lesions were graded according to U.S. BI-RADS criteria [19].

### 2.4. Ultrasound-Guided Biopsy

On all lesions with an overall BI-RADS score of 4 or 5, and on some lesions with a BI-RADS score of 3, ultrasound-guided biopsies were performed following international guidelines [20]. In short, after discussing the procedure with the patient and after acquisition of a written informed consent, skin disinfection, intracutaneous and ultrasound-guided perilesional application of a local anaesthetic (Mepivacain hydrochloride 1%), and a small skin incision, an automatic 12G or 14G core needle biopsy system (HistoCore Automatic Biopsy System, BIP GmbH, Türkenfeld, Germany) was used to acquire five tissue specimens, which were then embedded in a 5% formalin solution for further embedding and analysis.

### 2.5. Histological Evaluation and Immunohistochemistry

Histopathological data were retrieved from the hospital’s clinical information system KIS PowerChart (Cerner, North Kansas City, MO, USA) after imaging data collection was complete. Histopathological and immunohistochemistry findings were taken primarily from full-resection reports or otherwise from CNB specimens. If any form of neoadjuvant therapy had been administered since initial diagnosis, the histopathological core needle biopsy reports were used instead. Similarly, lymph node status was determined from sentinel or full resection lymph nodes in cases of no neoadjuvant therapy. Otherwise, results from an initial core needle biopsy (if available) were used. In the absence of histologically proven lymph node metastasis, lymph node status was considered negative following the local tumour board consensus.

### 2.6. Maximum Elastographic Peritumoural Halo Depth (SEPH_max_) Measurement

Lesion dimensions were measured in two axes in B-mode images and automatically transferred to the accompanying elastogram window during initial imaging. SEPH_max_ was measured in our in-house PACS image viewer (Agfa Impax EE, Agfa, Mortsel, Belgium) by (1) drawing the lesion outline in the B-mode image to accurately assess the tumour contour, (2) transferring it to the corresponding SE image in the dual-view mode, and (3) measuring the maximum SEPH_max_ depth of visual discernible elastographic perilesional stiffening regardless of orientation yet following the shortest line from the SEPH contour to the tumour surface. If the tumour could not fully be covered by the elastography box due to size or superficial position, then the elastography box was placed to cover a peripheral tumour section and at least 2 cm of surrounding tissue.

### 2.7. Statistics

All data were stored in Microsoft Excel 16.16.21 (Microsoft; Redmond, WA, USA). The statistical software used was R within RStudio 2021.9.1 (RStudio PBC, Boston, MA, USA) and GraphPad Prism 9.3.1 (GraphPad Software LLC; La Jolla, CA, USA).

Descriptive statistics for all patients include demographic (age) and disease-related factors (tumour side, quadrant, maximum diameter, grade, and LN metastasis rate) as described above. Results include mean ± standard deviation (SD) and ranges in brackets or relative frequency (absolute values) in brackets.

The SEPH_max_ was compared between (a) BI-RADS 2, 3, and 5 (grouped by histopathologic results) and (b) tumour grades 1–3 (again grouped by histopathologic results) using a Kruskal–Wallis test with Dunn’s multiple comparison test.

The correlation between the covariates described above and SEPH_max_ were assessed by a linear regression analysis. Continuous predictors were mean-centred and scaled by 1 standard deviation (SD).

Continuous data of the groups were compared via an ordinary one-way ANOVA with a Holm–Sidak correction or Kruskal–Wallis test with Dunn’s post-test (in case of non-Gaussian distribution, assessed by a D’Agostino and Pearson test) to correct for multiple testing. If through log-transformation a Gaussian distribution could be achieved, data was transformed for analysis. Categorical variables were compared via pairwise Fisher’s exact test (in case of 2 × 2 tables) or a χ^2^ test. Statistical significance was considered for *p*-values < 0.05.

Malignant tumours were grouped by Ki67 ranges (low risk: 5% or lower, intermediate risk: 6–30%, high risk: 30% or higher) following the St. Gallen risk classification [21], and the average elastographic halo depth was calculated for each group. Youden’s index was used to determine the ideal cut-off to separate low-risk from intermediate/high-risk tumours.

## 3. Results

### 3.1. Patient Demographics and Lesion Characteristics

**Figure 2:**
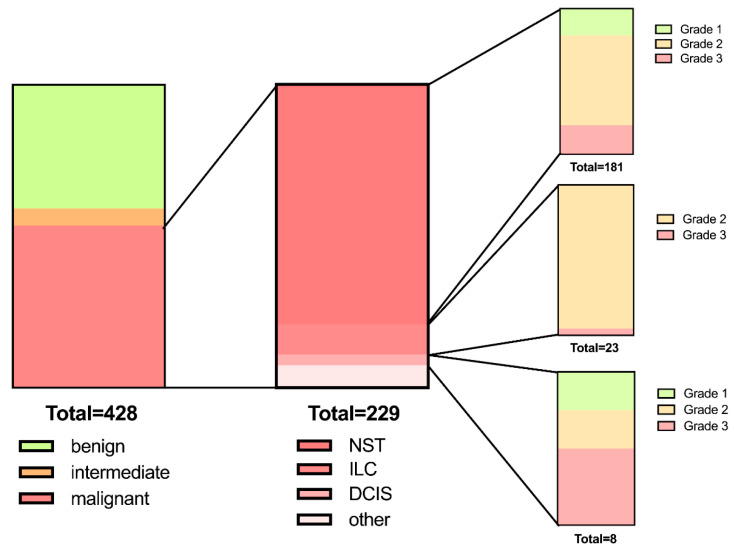
Overview of BI-RADS (**left**), tumour subtype (**middle**), and grade distribution of the main subtypes: no special type (NST), invasive lobular cancer (ILC), and ductal carcinoma in situ (DCIS) (**right**).

The average age of the 428 patients included was 56.0 ± 16.5 years. Of all 428 tumours, 40.9% (*n* = 175) were benign (histologically classified as BI-RADS II), 5.6% (*n* = 24) were of intermediate differentiation, and 53.5% (*n* = 229) were malignant. Among malignant tumours, grade 1 tumours constituted 23.8% (*n* = 43), grade 2 78.5% (*n* = 142), and grade 3 24.3% (*n* = 44). Lymph node metastasis was present in 18.3% (*n* = 42). The maximum tumour diameter was 15.4 ± 9.0 mm on average (range 2.9 to 57.3 mm). Lesions were mostly located in the upper outer quadrant (48.8%, *n* = 209), followed by the lower outer (19.2%, *n* = 82) and upper inner quadrant (16.6%, *n* = 71). For an overview, please refer to Table 2 and Figure 2.

### 3.2. Covariates for Maximum Elastographic Peritumoural Halo Depth

**Figure 3:**
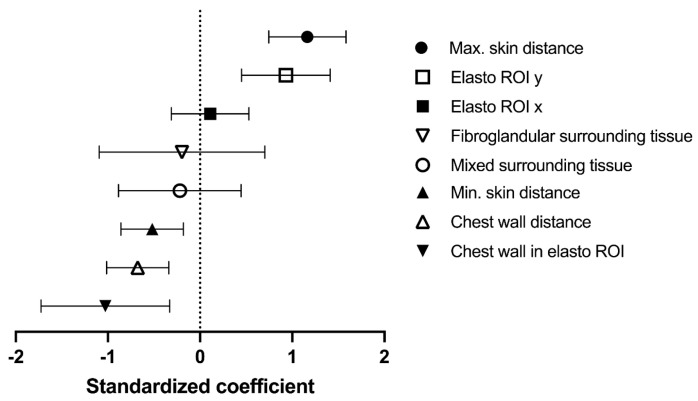
Standardized linear regression coefficients for the correlation with maximum strain elastographic peritumoural halo depth (SEPH_max_).

SEPH_max_ correlated positively with maximum skin distance (1.16 [95% CI 0.75 to 1.584], *p* < 0.001), as well as elastographic ROI y-dimensions (0.93 [95% CI 0.45 to 1.41], *p* = 0.002), and negatively with minimum skin distance (−0.52 [95% CI −0.86 to −0.18], *p* = 0.012), chest wall distance (−0.68 [95% CI −1.01 to −0.34], *p* = 0.001), and if a significant portion of the chest wall was in the elastographic ROI (−1.03 [95% CI −1.73 to −0.33], *p* = 0.016) (Figure 3).

### 3.3. Diagnostic Utility of SEPH_max_ Assessment

**Figure 4:**
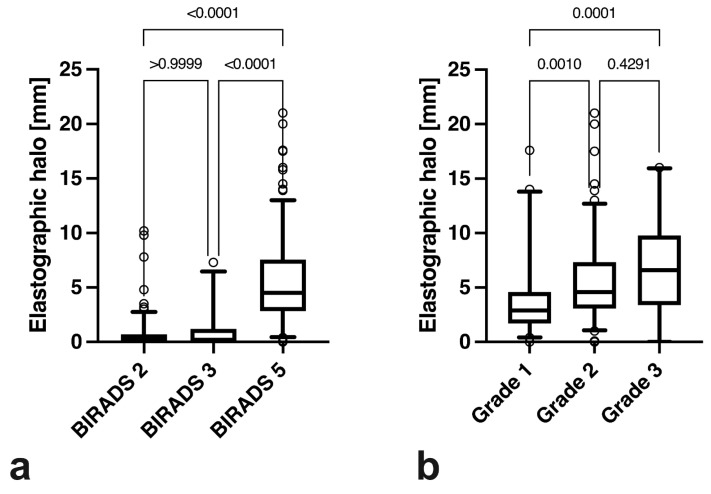
Comparison of maximum strain elastography peritumoural halo depth between BI-RADS II, III, and V lesions (**a**) and between BI-RADS V lesions with tumour grades 1, 2, and 3 (**b**).

Malignant tumours (5.5 ± 3.9 mm) had a significantly higher SEPH_max_ than intermediate (0.9 ± 1.7 mm, *p* < 0.0001) or benign breast lesions (0.6 ± 1.4 mm, *p* < 0.0001). No significant difference was encountered between benign and intermediate lesions (*p* > 0.9999) (Figure 4a). Furthermore, with increasing tumour grades, the average SEPH_max_ was significantly higher. While no significant difference was found between grades 2 and 3 (*p* = 0.43), grade 1 elastographic halo depth (4.0 ± 3.9 mm) was significantly lower than in grade 2 (5.6 ± 3.6 mm, *p* = 0.001) and grade 3 (6.8 ± 4.2 mm, *p* = 0.0001) (Figure 4b). Please also refer to Table 3.

**Figure 5:**
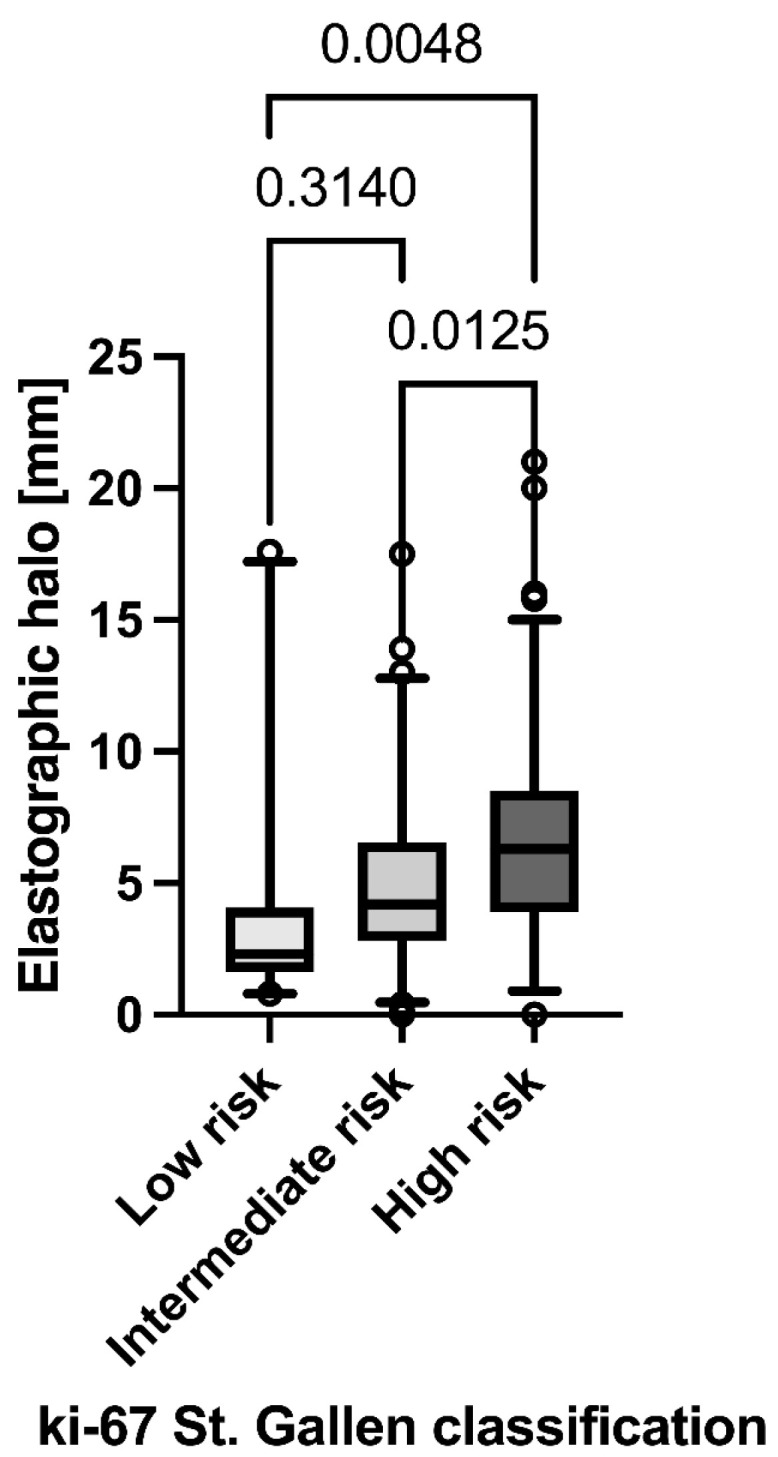
Maximum strain elastography peritumoural halo depth grouped by St. Gallen classes of Ki67 percentage (low risk: 5% or lower, intermediate risk: 6–30%, high risk: 30% or higher).

Furthermore, after Ki67 stratification, low-risk (*p* = 0.005) and intermediate-risk tumours (*p* = 0.013) had a significantly lower SEPH_max_ than high-risk tumours (Figure 5). Using a SEPH_max_ cut-off of 3.3 mm, identification of low-risk tumours showed 72.9% sensitivity and 73.8% specificity (ROC AUC 0.77 [95% CI 0.67 to 0.88], *p* < 0.0001).

### 3.4. Comparison of SEPH_max_ to Other Demographic and Sonographic Tumour Properties

**Figure 6:**
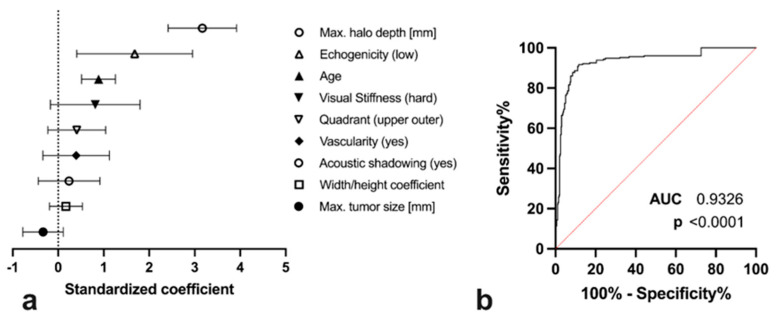
Standardized binary regression coefficients for the diagnosis of malignancy (**a**) and receiver-operating characteristic curve (**b**) for the differentiation between benign/intermediate and malignant breast tumours using the maximum halo depth at a cut-off of 2 mm.

Malignant lesion differentiation was significantly associated with SEPH_max_ (standardized regression coefficient 3.17 [2.42 to 3.92], *p* > 0.001), as were low lesion echogenicity (1.68 [95% CI 0.41 to 3.00], *p* = 0.030) and higher patient age (0.89 [95% CI 0.52 to 1.26], *p* > 0.001). Visual assessment of lesion stiffness (0.82 [95% CI −0.17 to 1.80], *p* = 0.173), affected quadrant (0.41 [95% CI −0.23 to 1.05], *p* = 0.292), tumour vascularity (0.40 [95% CI −0.34 to 1.13], *p* = 0.374), acoustic shadowing (0.24 [95% CI −0.44 to 0.92], *p* = 0.561), width/height coefficient (0.17 [95% CI −0.19 to 0.53], *p* = 0.442), and maximum tumour size (−0.33 [95% CI −0.78 to 0.11], *p* = 0.220) did not show a significant correlation (Figure 6a).

Based on a cut-off of 2.0 mm, SEPH_max_ demonstrated 87.8% sensitivity (95% CI 82.90% to 91.40%) and 90.5% specificity (85.57% to 93.80%) for the identification of a malignant breast lesion. The ROC AUC was 0.93 (*p* < 0.0001, Figure 6b).

## 4. Discussion

As described in the American College of Radiology’s Breast Imaging Reporting and Data System (BI-RADS) lexicon, several U.S. imaging features portend to a malignant and more aggressive breast-cancer phenotype; among those are irregular shape, angular or spiculated margins, antiparallel orientation, and posterior shadowing with or without an echogenic halo [22]. Furthermore, the higher the tumour grade—a generalized representation of biological tumour aggressivity, based on mitotic rates, nuclear polymorphia, and tubule formation—the higher the likelihood of suspicious imaging features, as illustrated among several studies on the topic, for example by Blaichmann et al. [23], which showed that grade III IDCs were more likely to present with microlobulated margins and posterior acoustic enhancement compared to lower-grade IDCs.

To improve the diagnostic accuracy of ultrasounds and more reliably identify lesions warranting further histological workup, elastography has seen increased use in breast ultrasounds in the past decade. While shear wave elastography has become more common in recent years [10], the mainstay of tissue elasticity assessment still is strain elastography, in which the examiner exerts repeated pressure on the tissue via the transducer, and the relative tissue compressibility is then calculated, leading to elastography maps [13]. The composition of the breast tissue is special in that it is the only organ in which focal lesions may show a difference in tumour size between B-mode and elastography, in contrast to organs such as the prostate and the thyroid [10]; a discrepancy between those two modes is especially frequent in malignant breast lesions, which can appear larger in SE maps than in the B-mode image, whereas benign breast lesions often have the same size or even appear smaller on elastography [24]. As many studies have focused on the central stiffness of a breast lesion or the elasticity imaging/B-mode ratios or other ratios, we put our main focus on the peritumoural tissue and the maximum halo depth.

A clear difference regarding the maximum extent of peritumoural stiffening between benign, intermediate, and malignant lesions could be shown. Furthermore, higher tumour grades were associated with greater maximum SE halo depth.

At the root of this peritumoural reduction in tissue elasticity, several interface processes between the host and the malignant tissue have been discussed [17], including periductal fibroelastic reactions, peritumoural infiltration, and reactive inflammation [18,25]. Non-invasive tumours such as ductal carcinoma in-situ (DCIS) and invasive mucinous carcinomas, on the other hand, may lack peritumoural stiffening, reflecting their absence or a low degree of surrounding tissue invasion [26].

**Figure 7:**
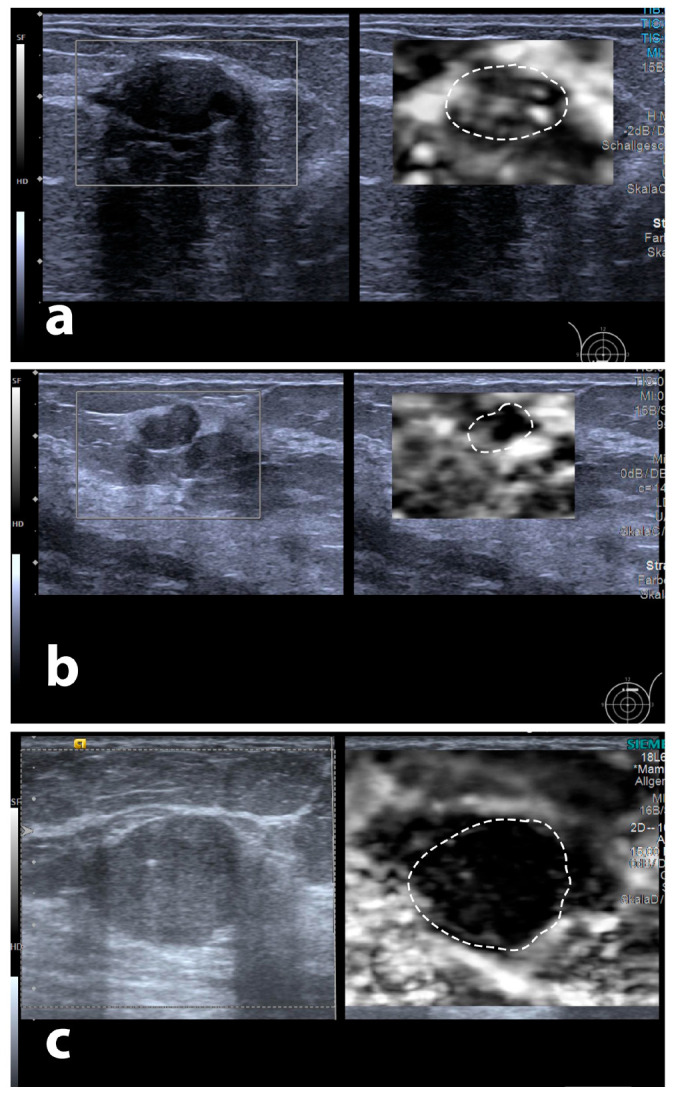
Examples for differing degrees of maximum peritumoural halo depth, three tumours with B-mode echotexture and surface: (**a**) BI-RADS 2 lesion (fibrous-cystic mastopathy) in a 45-year-old patient, (**b**) BI-RADS 3 tumour (atypical ductal hyperplasia) in a 21-year-old patient, and (**c**) BI-RADS 5 tumour (NST G2) in a 58-year-old patient. Dotted contours represent tumour outlines transferred onto elastography images.

In contrast to Zhou et al. [18], we found no association of tumour size and maximum halo depth, making it an independent factor. Therefore, assessment of the maximum SE halo depth may enable radiologists to more reliably identify those newly found lesions warranting further biopsy and to quickly estimate a newly found tumour’s biological aggressivity (for illustrative cases, please refer to Figure 7). This potential appears true even in very small or large tumours, according to our data. This approach may lead to a decrease in unnecessary biopsies, as follow-up examinations may suffice based on multiparametric ultrasound findings, as described before [27]. Additionally, it may be easier to argue for a re-biopsy in cases with biopsy results contradicting imaging findings. Increased suspicion on the basis of the imaging features would be valuable information for the interpreting pathologist, as differentiation between certain grades of breast cancer can be histologically difficult, depending on the quality of tissue specimens, and can vary among pathologists [28,29].

Besides the significant correlation of malignant lesion differentiation and the maximum SE halo depth, the evaluation of the halo depth may correlate with further tumour properties such as the Ki67 index. We could demonstrate that high-risk tumours with a Ki67 value > 30% have a significantly higher strain halo depth (cut-off for low-risk patients with Ki67 < 5% was <3.3 mm halo depth). According to the St. Gallen international consensus guidelines [21], systemic chemotherapy is indicated for breast lesions with Ki67 > 30% [30]. Accordingly, a very high SEPH_max_ should raise the suspicion of an aggressive tumour variant.

Besides SEPH_max_, which correlated significantly with malignant lesion differentiation, low lesion echogenicity and increased patient age were significant diagnostic markers for the differentiation of benign and malignant entities, in line with previous literature [23]. Visual assessment of lesion stiffness, affected quadrant, tumour vascularity, acoustic shadowing, width/height coefficient, and maximum tumour size did not show a significant correlation with tumour differentiation in our multiparametric analysis, however.

### Limitations

**Figure 8:**
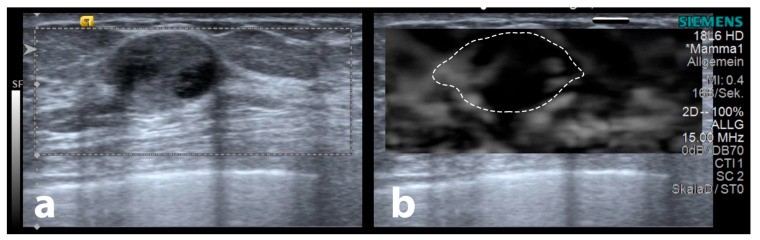
Sample of an encapsulated papillary carcinoma in the B-mode (**a**), demonstrating no surrounding strain elastography halo (**b**). The dashed outline demonstrates the lesion’s B-mode extent.

There are some limiting factors to this study. Firstly, the study was done in a single imaging centre with only three models of ultrasound machines from the same vendor; therefore, multi-centre, larger scale studies with a variety of ultrasound machines should be conducted to rule out vendor and centre-dependent biases and potentially identify improvements to our approach. Secondly, SE is known to be user dependent, which may influence results based on applied pressure and relaxation, potentially influencing halo results. Third, some entities such as encapsulated papillary carcinomas (EPC) with a low invasive potential and lower production of invasion-associated markers (such as matrix metalloproteinases, transforming growth factor receptor-beta, vascular endothelial growth factor [VEGF], and E-cadherin) [31,32] may not show a peritumoural halo (also refer to Figure 8). Additionally, care must be taken when measuring the SE halo depth, as it may be influenced by the maximum skin distance, the distance to the chest wall, and whether a significant portion of the chest wall was included in the elastographic ROI, and this influence can lead to measurement errors. Finally, mammographic findings were not analysed. Further studies should focus on whether similar peritumoural changes may aid in the diagnosis of newly-diagnosed breast lesions.

## 5. Conclusions

Assessment of SEPH_max_ appears to be a promising diagnostic tool in the classification and characterisation of newly diagnosed breast tumours, since the SEPH_max_ was significantly higher in BI-RADS 5 tumours (5.5 ± 3.9 mm) compared to BI-RADS 3 (0.9 ± 1.7 mm) and BI-RADS 2 (0.6 ± 1.4 mm). Furthermore, the assessment of the peritumoural halo allows conclusions to be drawn about the underlying tumour properties such as the Ki67 value. Low-risk tumours have a significantly lower SEPH_max_ compared to intermediate and high-risk tumours with a cut-off value of 3.3 mm. Therefore SEPH_max_ may offer a tumour-size-independent preliminary assessment of tumour aggressiveness and may enable radiologists to identify lesions warranting observation rather than immediate biopsy. It has several advantages over other imaging techniques, including non-invasiveness, availability, and short acquisition time. Further research is needed to validate these findings and to determine the optimal protocol for peritumoural halo measurement in breast cancer diagnosis and management.

## Figures and Tables

**Table 1:** Inclusion and exclusion criteria.

Inclusion Criteria	Exclusion Criteria
Ultrasound-guided biopsy of a newly diagnosed lesion with definable borders	Patient age under 18 years
Sufficient ultrasound documentation including B-mode, Doppler, and elastography imaging	Prior history of ipsilateral breast cancer
Available histological report	Insufficient ultrasound documentation
	Inconclusive histology

**Table 2:** Patient and lesion characteristics.

	Overall	BI-RADS II	BI-RADS III	BI-RADS V	*p*-Value ^$^
*n*	428	175	24	229	n/a
Age [years]	56.0 ± 16.5	48.3 ± 14.8	54.2 ± 16.3	62.2 ± 15.3	0.284/<0.0001/0.066 ^#^
Side, right (%/*n*)	47.9 (205)	48.6 (85)	25.0 (6)	49.8 (114)	0.067 ^§^
Maximum diameter (mm)	15.4 ± 9.0	14.9 ± 9.4	16.4 ± 10.8	15.7 ± 8.6	>0.999/0.316/>0.999 ^#^
Grade 1/2/3 (%/*n*)	-	-	-	G1: 23.8% (43) G2: 78.5% (142) G3: 24.3% (44)	n/a
Quadrant UI/ LI/ LO/ UO/ C (%/*n*)	16.6% (71)/ 8.9% (38)/ 19.2% (82)/ 48.8% (209)/ 6.5% (28)	17.1% (30)/ 9.7% (17)/ 21.7% (38)/ 42.3% (74)/ 9.1% (16)	12.5% (3)/ 12.5% (3)/ 16.7% (4)/ 41.7% (10)/ 16.7% (4)	16.6% (38)/ 7.9% (18)/ 17.5% (40)/ 54.6% (125)/ 3.5% (8)	0.076 ^§^
Lymph node metastasis (%/*n*)	-	-	-	18.3% (42)	n/a

^$^ Group comparison between BI-RADS II & III, II & V, and III & V, UI: upper inner, LI: lower inner, LO: lower outer, UO: upper outer, C: central/retromamillary; ^#^ Kruskal–Wallis with Dunn’s post-test; ^§^ χ^2^ test.

**Table 3:** Maximum strain elastography peritumoural halo depth by BI-RADS (upper row) and tumour grade (lower row).

BI-RADS	Benign (BI-RADS II)	Intermediate (BI-RADS III)	Malignant (BI-RADS V)	*p*-Values ^$^
SEPH_max_	0.6 ± 1.4 mm	0.9 ± 1.4 mm	5.5 ± 3.9 mm	>0.9999/<0.0001/<0.0001
Tumour grade	Grade I	Grade 2	Grade 3	*p*-values ^$^
SEPH_max_	4.0 ± 3.9 mm	5.6 ± 3.6 mm	6.8 ± 4.2 mm	0.001/0.0001/0.4291

^$^ Comparison of left–middle, left–right, and middle–right columns.

## Data Availability

Data will be available on request to authors.

## Abbreviations

The following abbreviations were used in this manuscript:

SE	strain elastography
SEPH_max_	maximum strain elastography peritumoural halo
NST	no special type
ILC	invasive lobular cancer
DCIS	ductal carcinoma in situ
US	ultrasound
ROI	region-of-interest
